# Statistical Approaches for Gene Selection, Hub Gene Identification and Module Interaction in Gene Co-Expression Network Analysis: An Application to Aluminum Stress in Soybean (*Glycine max* L.)

**DOI:** 10.1371/journal.pone.0169605

**Published:** 2017-01-05

**Authors:** Samarendra Das, Prabina Kumar Meher, Anil Rai, Lal Mohan Bhar, Baidya Nath Mandal

**Affiliations:** 1 Division of Statistical Genetics, ICAR-Indian Agricultural Statistics Research Institute, New Delhi, India; 2 Centre for Agricultural Bioinformatics, ICAR-Indian Agricultural Statistics Research Institute, New Delhi, India; 3 Division of Design of Experiments, ICAR-Indian Agricultural Statistics Research Institute, New Delhi, India; Chinese Academy of Sciences, CHINA

## Abstract

Selection of informative genes is an important problem in gene expression studies. The small sample size and the large number of genes in gene expression data make the selection process complex. Further, the selected informative genes may act as a vital input for gene co-expression network analysis. Moreover, the identification of hub genes and module interactions in gene co-expression networks is yet to be fully explored. This paper presents a statistically sound gene selection technique based on support vector machine algorithm for selecting informative genes from high dimensional gene expression data. Also, an attempt has been made to develop a statistical approach for identification of hub genes in the gene co-expression network. Besides, a differential hub gene analysis approach has also been developed to group the identified hub genes into various groups based on their gene connectivity in a case *vs*. control study. Based on this proposed approach, an R package, i.e., dhga (https://cran.r-project.org/web/packages/dhga) has been developed. The comparative performance of the proposed gene selection technique as well as hub gene identification approach was evaluated on three different crop microarray datasets. The proposed gene selection technique outperformed most of the existing techniques for selecting robust set of informative genes. Based on the proposed hub gene identification approach, a few number of hub genes were identified as compared to the existing approach, which is in accordance with the principle of scale free property of real networks. In this study, some key genes along with their Arabidopsis orthologs has been reported, which can be used for Aluminum toxic stress response engineering in soybean. The functional analysis of various selected key genes revealed the underlying molecular mechanisms of Aluminum toxic stress response in soybean.

## Introduction

With the advent of fast and cheaper genome sequencing technologies, huge genomic data is being generated and deposited in public domain databases over the years by different research organizations across the globe [1, 2]. Most of these datasets are related to expression of genes from various experiments conducted to understand behavior of biological mechanism of species under biotic and abiotic stresses. In due course of time huge gene expression data is generated through microarray experiments under these stresses. Integration and analysis of data generated by microarray experiments for the same stress or related conditions is essential to enhance the sensitivity of the hypothesis under consideration for drawing valid conclusions [3]. For instance, meta-analysis of microarray data pertaining to different experiments in rice and *Arabidopsis* revealed the presence of highly connected key genes that are central to the plant defense system under various biotic and abiotic stresses [4, 5].

Usually, microarray data are used for gene selection and modules detection in genetic network analysis, which suffers from the inherent limitation of its high dimensionality, *i*.*e*. the number of genes is much larger than the number of subjects/samples [6]. Therefore, it is important to select most relevant genes related to stresses/conditions from thousand(s) of genes with the help of appropriate computational approach(s). In this regard, Volcano plot method [7] is quite popular among the researchers in which genes are selected by considering their relevance with their classes. However, such method may not be sufficient to discover some complex relationships among genes for a certain trait or condition [8]. Besides, several statistical and machine learning methods, *viz*. t-score, F-score, Information Gain (IG) measure, Random Forest (RF) and Support Vector Machine-Recursive Feature Elimination (SVM-RFE) [7, 9–13] have also been used for gene selection. However, in these methods genes are selected by considering only their relevance with classes. In such case, there is a possibility that genes which are spuriously associated with the classes may also get selected.

In order to understand the interrelationship among the selected genes, identification of gene modules and key genes responsible for a particular stress/condition, analysis of gene co-expression networks need to be carried out. Weighted Gene Co-expression Network Analysis (WGCNA) [14] is a latest and popular technique used to decipher co-expression patterns among genes. The WGCNA approach typically deals with the identification of gene modules by using the gene expression levels that are highly correlated across samples [14]. This technique has been successfully utilized to detect gene modules in *Arabidopsis*, rice, maize and poplar for various biotic and abiotic stresses [5, 15–18]. Further, this approach also leads to construction of Gene Co-expression Network (GCN), a scale free network, where, genes are represented as nodes and edges depict associations among genes [14, 19]. In such network, highly connected genes are called hub genes, which are expected to play an important role in understanding the biological mechanism of response under stresses/conditions [20–24]. Identification of hub genes will also help in mitigating the stress in plants through genetic engineering. The existing approaches [21–24] have mainly focused on hub gene identification, based only on gene connection degrees in the GCN. Moreover, these techniques select such genes empirically without any statistical criteria. Besides, few approaches can be found in the literature for the identification of hub nodes in a scale free network [22–24].

Aluminum (Al) toxic stress is a major impediment to the crop production on acidic soils that affects about 30–40% of the world’s arable lands [25]. Soybean (*Glycine max* L.), that provides major source of proteins, unsaturated fats, carbohydrate and fibers, is one of the most important legume crop, capable of providing nutritional security to the global population. Soybean is preferably grown on acidic soil and its productivity is significantly reduced by Al toxic stress. In acidic soil, Al stress causes rapid inhibition in root growth and subsequently inhibits water and nutrient uptake by plants. This increases the susceptibility of plants to other environmental stresses and results in reduction of crop productivity [26]. Under heavy pressure of population explosion and global warming, achieving nutritional security in general and protein security in particular through enhancing the productivity of soybean is of paramount importance. However, the underlying mechanisms for Al toxic stress response in plants in general and soybean in particular are not so clearly deciphered till now [27].

In this study, a statistical technique *i*.*e*. Bootstrap SVM-RFE (Boot-SVM-RFE) is proposed for selection of informative genes. In this technique, genes are selected after reducing the effect of spurious associations between genes and classes. The performance of the proposed gene selection technique is found to be better than the existing techniques, while compared by using three different datasets. Further, a statistical approach for identification of hub genes in the GCN was also proposed. Again, this approach is evaluated on the genes selected from the above datasets and found to be superior in terms of scale free property of biological network. Besides, an R package has been developed based on the proposed hub gene identification approach. Further, an attempt has been made to integrate and analyze the gene expression datasets generated by different experiments for the identification of Al toxic stress responsive genes in soybean, by using the proposed techniques. Hub genes responsible for Al toxic stress have been identified and their functional analysis has been done.

## Materials and Methods

The soybean microarray experimental datasets under Al stress were collected from Gene Expression Omnibus with platform GPL4592 (http://www.ncbi.nlm.nih.gov/geo/query/acc.cgi?acc=GPL4592). This platform contains 3855 experimental samples on 37,593 probes generated using Affymetrix Soybean Genome Array. Out of these samples, 80 samples related to Al stress were collected for further study. Initially, raw CEL files of these collected samples were processed using Robust Multichip Average (RMA) algorithm available in *affy* Bioconductor package of R [28–30]. This includes background correction, quantile normalization and summarization by the median polish approach [31]. Then the microarray experimental samples with mean ≥ 7.1 and standard deviation ≤ 2.5 were selected, as uniformity of colors in the correlation plot was observed for these parameters setting (S1 Fig). Through this process, 78 samples (generated over 3 different experiments) were selected. The descriptions about the selected samples are given in S1 Document. The log2 scale transformed expression data from the RMA for these selected experimental samples were used for further statistical analysis.

### Bootstrap support vector machine- recursive feature elimination technique (Boot-SVM-RFE)

Here, we propose a technique *i*.*e*. Boot-SVM-RFE for selection of informative genes from high dimensional gene expression dataset. In this approach, a Non-Parametric (NP) hypothesis testing procedure was used for the identification of informative genes based on their statistical significance. Earlier, SVM-RFE method was used for ranking of genes from gene expression data for identification of cancer responsible genes [13]. In this algorithm, genes are individually eliminated based on their least significance in classification during SVM training. The objective function, *J* for this classification problem is defined as:

$$J={\left\|w\right\|}^{2}/2$$ (1)

where, *w* is kernel width computed by SVM. The Optimal Brain Damage algorithm [32] was used to approximate the change in *J*, after deletion of *i-th* gene from the dataset. Further, expanding *J* (up to second order) with the help of Taylor series approximation [6], the value of *J* was given by

$$\Delta J(i)=(1/2)\frac{\partial^{2}J}{\partial w_{i}^{2}}{\left(\Delta w_{i}\right)}^{2}$$ (2)

where, Δ*w*_*i*_ is change in weight due to removing *i-th* gene from existing dataset and Δ*J*(*i*) is used as weight pruning criterion.

It may be noted here that, the cost function *J* is a quadratic function of *w*_*i*_ and both are directly proportional to each other. Hence, measurement of either *w*_*i*_ or *J* provides equivalent information. Keeping this in view $w_{i}^{2}$ is used as the ranking criterion for evaluating impact of *i-th* gene on this classification [6]. In this process, genes are eliminated with the smallest $w_{i}^{2}$ iteratively in a backward elimination manner and ranked gene list is prepared at the end. Moreover, most of the gene selection methods are sensitive to small permutation of experimental conditions [13]. The ranking of genes based on high dimensional expression data may also lead to the selection of spurious genes and make the selection process unreliable [33]. Therefore, it is essential to select genes based on statistical testing instead of their ranks. Keeping in view the above fact, a test statistic has been proposed for selecting informative genes.

In this testing procedure, *n* bootstrap samples each of size *m* are selected randomly with replacement to construct a training set for SVM from available *M* samples in a dataset. Then SVM-RFE procedure was applied to each of these *n* bootstrap samples to get *n* list of genes along with their ranks. Therefore, each of genes will have *n* number of ranks (one for each bootstrap). Let a score function *i*.*e*. *Rank Score* (*R*_*ij*_) is defined to convert these ranks of each gene into corresponding score in each bootstrap sample, as

$$R_{ij}=\frac{N+1-p_{ij}}{N}$$ (3)

where, *N* represents total number of genes considered in the dataset and *p*_*ij*_ (1 ≤ *p*_*ij*_ ≤ *N*) is the ranked position of *i-th* gene in *j-th* bootstrap sample. After getting the rank scores of all genes over *n* bootstrap samples, following proposed hypothesis needs to be tested for selection of informative genes.

$$\begin{array}{l} H_{0}:\;i-th\text{ gene is not informative }(i.e.\;R_{i}\leq \text{Q)}\\ H_{1}:i-th\text{ gene is informative }(i.e.\;R_{i}>\text{Q)} \end{array}$$

where, Q be the second quartile.

For *i-th* gene, *R*_*j*_^(*i*)^ (*N*^*-1*^ ≤ *R*_*j*_^(*i*)^ ≤ 1) is the rank score for *j-th* bootstrap sample (*j* = 1, 2, …, *n*). Further, *R*_*j*_^(*i*)^ is a random variable (*rv*). Since, *R*_*j*_^(*i*)^ is a function of rank, therefore, its empirical distribution is symmetric about the second quartile. So, without loss of generality, we defined another variable *r*_*j*_ (for fixed *i*) as:

$$r_{j}=R_{j}^{\left(i\right)}-Q$$ (4)

In order to test the statistical significance (*H*_0_ *vs*. *H*_*1*_) for gene *i*, the *r*_*j*_’s are arranged in ascending order of their magnitude and subsequently, the ranks *1*, *2*, *…*, *n* are assigned, keeping in mind their original signs. Let *T*^+^ be the sum of the ranks of positive *r*_*j*_’s and *T*^−^ be the sum of the ranks of negative *r*_*j*_’s. Now for finding distribution of test statistic *T*^+^, another variable *Z*_(*k*)_ is defined as:

$$Z_{\left(k\right)}=\left\{ \begin{array}{l} 1\text{ if the }\left|r_{j}\right|\text{has rank }k\;(>0\text{)}\\ 0\text{ else} \end{array}\right.$$ (5)

Here, *k* = {1, 2, …*n*}. Now, the variables *Z*_(*k*)_ are independent Bernouli variates and its order of moments can be obtained as:

$$E(Z_{\left(k\right)})=\frac{n}{2}\left(\begin{array}{l} n-1\\ k-1 \end{array}\right)\;B(k,\;n-k+\text{1)}$$ (6)

$${\text{Var }(\text{Z}}_{\text{(}k\text{)}}\text{)}=E\left\{Z_{\left(k\right)}(1-E(Z_{\left(k\right)}))\right\}$$ (7)

Then, the first two moments of the statistic (*T*^+^) can be written as:

$$E(T^{+})=\sum_{k=1}^{n}kE(Z_{\left(k\right)})$$ (8)

$$\text{Var}(T^{+})=\sum_{k=1}^{n}k^{2}\{E(Z_{\left(k\right)})(1-E(Z_{\left(k\right)}))\}$$ (9)

Let *R*_*i*_ be expected rank score for *i-th* gene over all bootstrap samples. Under the simple null hypothesis *H*_0_: *R*_*i*_ = Q against *H*_1_: *R*_*i*_ > Q the expressions in Eqs 8 and 9 can be written as

$$E_{H_{0}}(T^{+})=\frac{1}{2}\sum_{k}k=\frac{n(n+1)}{4}$$ (10)

$${\text{Var}}_{H_{0}}(T^{+})=\frac{1}{4}\sum_{k}k^{2}=\frac{n(n+1)(2n+1)}{24}$$ (11)

As the number of bootstrap samples are quite large, then under Linberg’s cental limit theorem [34, 35], *T*^+^ follows normal distribution asymptotically, *i*.*e*.

$$\frac{T^{+}-E_{H_{0}}(T^{+})}{\sqrt{{\text{Var}}_{H_{0}}(T^{+})}}\sim N(0,\;1)$$ (12)

Based on test statistic (Eq 12) under *H*_*0*_, it can be tested that whether a gene is informative or not. This procedure was repeated for other genes. The performance of the proposed technique for selection of informative genes was compared with respect to existing techniques *viz*. SVM-RFE, t-score, F-score, RF and IG on gene expression data of Al stress in soybean, salinity and cold stress in rice (Table 1). In order to assess the performance of genes selection techniques, the fixed number of top ranked genes selected through these techniques were used in SVM classifier to discriminate the class labels of samples between stress (+1) and control (-1). In this case, SVM-Classification Accuracy (CA) was computed through a sliding window size technique. Here, the window sizes (number of sorted genes) were taken as 50, 100, …, 500 with sliding length of 50. The performance of these techniques was adjudged on the basis of CA and it’s co-efficient of variation (CV).

**Table 1:** Microarray studies used in comparative analysis.

Data descriptions	#GEO Series	# Genes	# Samples	# Classes
Salt stress in Rice	6	6637	70	2 (stress: 1 and control: -1)
Cold stress in Rice	5	8839	100	2 (stress: 1 and control: -1)
Al stress in Soybean	4	15510	68	2 (stress: 1 and control: -1)

# GEO series: Number of GEO series; # Genes: Number of genes; #Samples: Total number of microarray samples; # Classes: Number of classes

### Gene co-expression network analysis

GCNs were constructed by using gene co-expression measure that depicts association among genes [23, 36]. Let ***x***_*i*_ be the expression profile of *i-th* gene, *i*.*e*. the expression values of *i-th* gene across all the microarray samples. Then, gene co-expression similarity measure *s*_*ij*_ between *i-th* and *j-th* gene is computed as the absolute value of Pearson’s Correlation Co-efficient (PCC) [14, 23], which is given by:

$$s_{ij}=\left|cor\;(x_{i},\;x_{j})\right|\;\forall \;i\neq j=1,\;2,\;\ldots ,\;G$$ (13)

The adjacency score (*a*_*ij*_) between *i-th* gene and *j-th* gene is defined in terms of *s*_*ij*_ [18] as:

$$a_{ij}=s_{ij}{}^{\beta }$$ (14)

where, *β* (≥1) is soft threshold power, determined by using the concept of scale free property of biological networks [23]. The detail methodology for determination of the soft threshold power has been discussed elaborately by Zhang and Horvath (2005) [14]. This soft threshold approach leads to a weighted GCN that satisfies the scale free property of biological networks. For both Al stress and control conditions, the value of *β* was taken as 8 for calculation of adjacency score (S2 Fig), with best approximation to scale free criteria [36] using *R*^*2*^ > 0.80 through fitting of Power law model. In order to identify the gene modules (*i*.*e*. group of tightly co-expressed genes) within the selected informative genes, the Topological Overlap Matrix was constructed based on the adjacency scores [14]. The *BlockWiseModules* function available in *WGCNA* package [19] of *R* was executed to identify these modules. For this purpose, various parameters like module size, deep split level and tree merge cut height was set at 20–30, 4 and 0.15–0.25 respectively. In order to find the consensus modules showing co-expression patterns of genes across stress and control conditions, the function *blockwiseConsensusModules* was used with parameter settings 8, 30 and 0.15 as power, minimum module size and merge cut height respectively.

### Proposed statistical approach for identification of hub genes

In network theory, a node is defined as hub node [20–24], if its connection degree is greater than average connection degree of the network [21]. In the existing approach, a gene is declared as hub gene based on an indicator function [21, 23], *i*.*e*. *Hub*_*i*_ = [*I*(ki > *τ*)] and number of hub genes (*NHub*) in the genetic network is calculated as $NHub=\sum_{i}\left[I(k_{i}>\tau )\right]$, where, *Hub*_*i*_: hub status of *i-th* gene (*i*.*e*.1 or 0); *k*_*i*_: connection degree of *i-th* gene; *τ*: threshold value *i*.*e*. average connection degree of the network. This technique selects hub genes empirically based on only observed gene connectivity without taking into account any statistical consideration. Therefore, an alternate statistical approach based on statistical significance of gene connectivity was proposed for detection of hub genes in the GCN. The proposed statistical approach is described as follows:

The Weighted Gene Score (WGS) for *i-th* gene in terms of weighted gene connectivity (*a*_*ij*_) can be written as:

$$WGS_{i}=\sum_{j}a_{ij}\;\forall \;i\neq j=1,\;2,\;\ldots ,\;G$$ (15)

where, *WGS*_*i*_ represents the relative importance of *i-th* gene based on its connections to the remaining genes in GCN. For the purpose of hub gene identification, following hypotheses are constructed.

$$H_{0}:\;WGS_{i}\leq \mu \;i.e.\;i-th\text{ gene in the GCN is not a hub gene}$$

$$H_{1}:\;WGS_{i}>\mu \;i.e.\;i-th\text{ gene in the GCN is a hub gene}$$

where, *μ* is average connection degree of the complete network model. Here in order to get the distribution of the test statistic under *H*_*0*_, a resampling procedure was used. In this procedure, *m* microarray samples were selected randomly with equal probability from *M* microarray samples to construct one subsample (for one GCN) (*m* ≤ *M*). Then statistical measures (Eqs 13–15) were applied to get WGS for each gene in that GCN. This procedure was repeated large number of times say *S* to get *S* sets of WGS. In this study, *S* = 500 was taken to get 500 random GCNs under stress and control conditions separately. For testing *H*_*0*_ *vs*. *H*_*1*_, a NP test statistic was proposed to test significance of the WGS for each gene, *i*.*e*. for testing whether WGS of a gene is greater than the average connection degree of the complete network or not. The proposed procedure for testing the hypothesis is as follows:

Let for a particular gene (*i*), *WGS*_*k*_^(*i*)^ be the WGS for *k-th* subsample (*k* = *1*, *2*,*…*, *S*). Here *WGS*_*k*_^(*i*)^ ‘s are *rvs*. So, without loss of generality, another variable *X*_*k*_ can be defined as:

$$X_{k}=WGS_{k}^{\left(i\right)}-\mu$$ (16)

In order to test the statistical significance of connectivity for gene *i*, the *X*_*k*_’s are arranged in ascending order of their magnitude and subsequently, the ranks *1*, *2*, *…*, *S* are assigned, keeping in mind the original signs of *X*_*k*_. Let *W*^+^ be the sum of the ranks of positive *X*_*k*_s and *W*^‒^ be the sum of the ranks of negative *X*_*k*_s. The distribution of the test statistic (*W*^+^) under *H*_*0*_ can be obtained by following the above approach to get the distribution of *T*^+^ in Boot-SVM-RFE. Further, under large number of subsamples (*S* = 500), following the central limit theorem the distribution of *W*^*+*^ is approximately standard normal, *i*.*e*.

$$\frac{W^{+}-E_{H_{0}}(W^{+})}{\sqrt{{\text{Var}}_{H_{0}}(W^{+})}}\sim N(0,\;1)$$ (17)

The procedure was repeated for each gene in the GCN and the statistical test was applied to identify hub gene based on significance values for both control and stress conditions separately.

**Algorithm**:

Step 1: Begin with all genes (nodes) in the GCN

Step 2: Construct a data set say *T*_*k*_ with *m* samples randomly taken from *M* microarray samples

Step 3: Calculate WGS for all genes

Step 4: Repeat Step 2 and 3 *S* times to get *S* sets of WGS for each gene

Step 5: Take a particular gene (*i-th* gene) along with its WGS

Step 6: Test the hypothesis for *i-th* gene and obtain its *p*-value

Step 7: Repeat the Step 5–6 for all genes (*i* = 1, 2, …, *G*)

Step 8: Rank the *p*-values and select the hub genes

The proposed hub gene identification approach for the GCNs constructed under two contrasting conditions (stress *vs*. control) can be called as Differential Hub Gene Analysis (DHGA). By this approach, the identification of hub genes is possible in both these GCNs based on statistical test of significance. On the basis of *p*-values, genes in the GCNs under either condition can be grouped into various groups, *viz*. Housekeeping Hub Genes (HHG), Unique Hub Genes (UHG) for stress, UHG for control, Non-hub genes based on a decision matrix (Table 2).

**Table 2:** Decision matrix for differential hub gene analysis.

Sl. No.	Stress Condition	Control Condition	Descriptions
1	*p* value < α	*p* value < α	Housekeeping hub gene
2	*p* value < α	*p* value > α	Unique hub gene for stress condition
3	*p* values > α	*p* value < α	Unique hub gene for control Condition
4	*p* value > α	*p* value > α	Not a hub gene

*p-value*: Obtained statistical hub gene significance value; α: Desired level of statistical significance

### Modeling of Module Interaction Network

A Module Interaction Network (MIN) can be defined as a digraph, where modules are nodes or units and edges depict regulatory relationships among the modules. Most of the approaches available in literature for modelling of genetic networks are not applicable to the gene expression data due to the problem of high dimensionality [37]. It is biologically required to consider gene module as a functional unit [18, 20]. Further, these modules can be taken as unit to study the interaction among the gene modules. The expression levels of the modules can be calculated as:

Md(t)=∑Gene i∈ module dGi(t)nd        where   1≤d≤D,         1≤t≤T (18)

where, *M*_*d*_ (*t*): expression level of *d-th* module (*d* = 1, 2,…, *D*) at time *t* (*t* = 1, 2, …, *T*), *G*_*i*_ (*t*): expression level of *i-th* gene at time *t*, *n*_*d*_: number of genes present in *d-th* module.

For Al stress data, all the selected microarray samples obtained through meta-analysis belong to time series experiments (the gene expression values were measured over 5 time points *e*.*g*. 0, 2, 12, 48 and 72 hours) (S1 Document). Further, the bspline method of data interpolation was used to interpolate the module expression values up to 50 time points in the interval of [0, 72 hours]. Then, we modeled the expression level of module *d* at time *t* as a linear regression with the expression levels of other modules at tim*e* (*t−1*). The model which depicts the interaction between these modules can be written as:

$$M_{d}(t)=\beta_{0}+\sum_{d\neq h}\beta_{h}M_{h}\;(t-1\text{)}+\varepsilon$$ (19)

where, the *β*_*h*_ s are regression coefficients and *ε* is the random noise with mean 0 and variance σ^2^. To compute the regression coefficients, which depicts the interaction among the modules, Bayesian Model Averaging (BMA) [38] algorithm was executed. Further, the posterior probabilities for each module interaction were calculated by using *iterative* BMA algorithm [39]. The module interactions were arranged in descending order by the value of posterior probabilities and significant module interactions were selected by fixing proper threshold. The MIN was constructed by using *RCytoscape* package [40].

## Results

### Performance analysis of Boot-SVM-RFE

In order to study the performance of the proposed Boot-SVM-RFE technique, the top 1000 genes obtained based on ranking from each of the gene selection techniques were used for classification of crop microarray samples into control and stress classes through SVM classifier. The CAs were measured for each sliding window size over 5 fold cross validation. The CAs for different sliding window sizes is given in Table 3. It is observed that for Al stress data, the CAs of Boot-SVM-RFE are higher than that of other techniques *viz*. SVM-RFE, t-score, F-score, RF and IG for the sliding window sizes 50, 100, 150, 200, 250 and 300. However, for higher window sizes *i*.*e*. 350, 400, 450 and 500, the CAs for Boot-SVM-RFE are at par with that of SVM-RFE but higher than that of t-score, F-score, RF and IG (Table 3). In general the performance of Boot-SVM-RFE is highest followed by SVM-RFE, RF, IG, F-score and t-score with respect to CA values for Al stress. However, in case of salinity and cold stress, the performance of Boot-SVM-RFE is observed to be better than other gene selection techniques irrespective of sizes of sliding window (Table 3). The order of the performance of different gene selection techniques in case of salinity stress is Boot-SVM-RFE > SVM-RFE > F score > RF > F score > t score > IG, whereas, in case of cold stress the order of performance is Boot-SVM-RFE > RF > IG > SVM-RFE > F score > t score (Table 3). From this performance analysis, it can be seen that the performance of the proposed Boot-SVM-RFE is consistently better over other contemporary techniques across different datasets related to abiotic stresses. Further, it is also observed that Boot-SVM-RFE has less CV for most of the sliding window sizes with respect to other five techniques when applied to the datasets of these three stresses (Table 3).

**Table 3:** Comparison of Boot-SVM-RFE with other competitive algorithms for different sliding window sizes.

	Boot-SVM-RFE		SVM-RFE		t-Score		F-Score		IG		RF	
WS	CA	CV	CA	CV	CA	CV	CA	CV	CA	CV	CA	CV
Aluminum stress gene expression data in Soybean												
50	95.629	2.622	93.421	2.778	89.127	5.342	89.820	4.081	90.859	3.146	92.105	3.719
100	96.199	2.926	92.249	4.297	90.789	4.008	91.667	3.303	92.251	3.910	92.471	3.929
150	96.279	3.020	94.362	3.215	90.480	2.386	91.950	3.501	92.337	4.341	93.040	2.889
200	97.724	2.182	96.135	2.619	90.378	3.748	91.776	3.608	94.408	4.594	93.572	2.584
250	96.737	2.356	93.544	2.905	91.404	2.767	91.667	4.260	93.070	2.417	93.860	3.461
300	97.086	2.203	95.335	2.770	91.635	3.845	91.447	3.775	94.549	3.489	95.771	2.861
350	97.862	2.606	97.470	2.431	91.397	4.904	92.915	4.150	94.737	4.049	94.737	3.586
400	97.930	1.842	97.368	1.911	92.982	2.031	93.311	2.974	94.627	3.998	95.724	2.563
450	97.249	2.599	97.129	2.332	93.062	2.009	92.943	3.541	95.096	2.239	95.813	2.934
500	97.763	2.011	97.632	2.273	93.289	3.669	93.421	3.814	94.342	4.314	96.316	3.075
Mean	97.046		95.464		91.454		92.092		93.627		94.340	
Salinity stress gene expression data in Rice												
50	97.218	1.927	94.015	3.382	90.000	3.346	93.684	4.498	90.150	5.200	93.684	2.401
100	98.175	1.203	96.984	1.742	92.778	2.613	94.444	2.690	92.222	3.242	94.841	2.375
150	98.319	0.924	95.731	1.402	92.773	3.054	95.378	1.874	93.697	2.474	95.462	2.065
200	98.482	0.832	96.786	2.052	93.571	2.493	95.804	2.071	93.304	1.651	95.446	2.363
250	98.190	1.162	97.810	1.218	93.333	2.432	96.286	2.157	93.333	2.432	95.905	1.856
300	98.265	0.842	97.449	1.742	94.490	3.015	96.653	1.244	93.265	2.118	96.327	1.813
350	98.352	0.545	96.923	1.455	95.055	1.693	96.692	1.419	93.187	1.421	96.154	1.407
400	98.571	0.000	96.619	1.151	94.167	2.543	97.143	1.659	94.286	2.238	95.952	1.533
450	98.571	0.000	97.273	1.386	93.636	2.399	97.922	1.197	94.416	1.258	95.714	2.111
500	97.000	1.465	96.857	1.942	95.000	2.270	97.000	2.018	94.286	1.428	95.286	1.742
Mean	98.114		96.645		93.480		96.101		93.215		95.477	
Cold stress gene expression data in Rice												
50	96.328	1.830	94.947	2.031	94.000	1.701	94.579	2.153	94.526	2.322	94.526	2.221
100	97.175	1.387	95.778	2.043	94.333	2.356	95.889	1.820	95.722	2.209	95.611	2.224
150	97.507	0.932	96.471	1.762	94.235	2.236	95.235	1.983	95.824	1.760	96.294	2.080
200	98.482	0.832	97.000	1.304	95.500	1.622	95.875	1.861	96.250	1.615	97.375	2.368
250	98.190	1.162	96.067	1.906	95.333	1.969	95.933	1.446	96.333	1.472	96.267	2.051
300	98.265	0.842	96.000	1.634	95.786	1.487	96.014	1.935	96.143	1.255	96.643	1.855
350	96.785	0.554	96.923	1.296	95.923	1.163	96.062	1.247	96.154	1.432	97.923	1.742
400	98.881	0.687	95.567	2.027	95.667	1.433	96.667	1.273	96.000	1.740	97.333	1.752
450	98.777	0.383	95.545	1.432	95.818	1.671	95.909	1.185	97.727	1.033	97.545	1.855
500	97.679	1.454	96.700	1.545	94.500	1.433	95.100	1.353	97.300	1.078	97.300	1.594
Mean	97.807		96.100		95.110		95.726		96.197		96.681	

Boot-SVM-RFE: Bootstrap SVM-RFE; RF: Random forest; IG: Information gain measure; WS: Sliding window Sizes; CA: Classification accuracy; CV: Co-efficient of Variation in CA

### Selection of informative genes for Al stress in soybean

**Fig 1:**
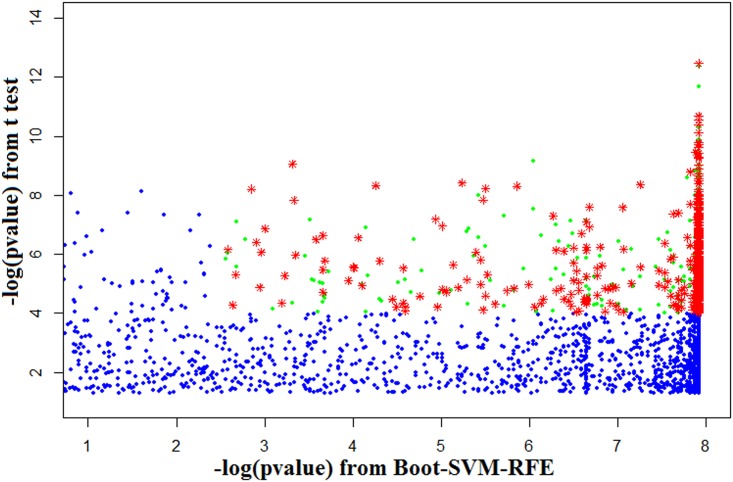
Gene selection plot for selection of informative genes for Al stress in soybean. The horizontal axis represents *negative logarithm of statistical significance values* obtained from Boot-SVM-RFE. The vertical axis shows the *negative logarithm of statistical significance values* from t-test. Green dots indicate selected probes with–log (*p-value*) from Boot-SVM-RFE ≥ threshold of 2.5 and t-test–log (*p-value*) ≥ threshold of 4. Red stars indicate the selected probes which have *Arabidopsis* orthologs. Blue dots indicate unselected probes.

Since the Boot-SVM-RFE was found to be superior as compared to other gene selection techniques, it was further employed to select informative genes for Al stress in soybean. In order to get a robust and minimal set of informative genes, the fold change in Volcano plot was replaced with–log10 (*p-values*) obtained from Boot-SVM-RFE and then a gene selection plot was constructed. The threshold values for Y and X-axis of the gene selection plot were fixed as 4 and 2.5 respectively, which lead to selection of 981 genes (Fig 1). The consensus sequences of these 981 genes obtained from GeneChip Soybean Genome Array of Affymetrix were then used to identify the *Arabidopsis* orthologs [41] and it was found that 554 genes have unique orthologs in *Arabidopsis* (Fig 1). Further, the annotations of these selected genes were obtained from SoyBase (http://soybase.org) [42]. A brief description about these selected genes is given in S1 Table.

### Functional analysis of selected genes for Al stress in soybean

**Fig 2:**
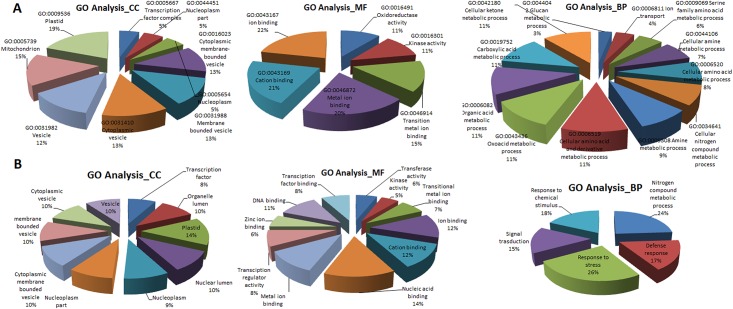
Functional enrichment analysis of selected genes and hub genes under Al stress. The GO term enrichment analysis of 981 selected informative genes (A) and hub genes (B) for Al stress condition using *Agrig*o is shown for different gene ontology categories (CC, MF and BP). For (A), the GO terms are chosen whose p-values < 0.008 and FDR values (false discovery rate) < 0.6. For (B), the GO terms are chosen whose p-values < 0.1 and FDR values < 0.8.

The Gene Ontology (GO) enrichment analysis of the 981 selected genes was performed by using *AgriGO* [43], a plant-specific GO term enrichment analysis tool. It is observed that most of the selected genes are responsible for transition metal ion binding, metal ion binding, cation binding, ion binding, *etc*. (Fig 2A). These molecular functions (MF) might be activated due to high concentration of Al ions in water or soil. Two other MF *i*.*e*. oxido-reductase (redox) and kinase activities are also present in these selected genes (Fig 2A). The significant behavior of the genes in redox activity might be related to electron transport in complex chemical reactions that balances the charges during ion transport. The redox activity might also be related to Reactive Oxygen Species (ROS) that are produced in response to oxidative stress due to water deficit during abiotic stress like Al toxic stress [44]. In biological process categories, such as cellular nitrogen compound metabolic process, amine metabolic process, cellular amino acid and derivative metabolic process, oxoacid metabolic process, organic acid metabolic process, carboxylic acid metabolic process, cellular ketone metabolic process and ion transport activity, the number of selected genes is more as compared to other biological processes (Fig 2A). It may be inferred that some of these chosen genes are involved in ion transport activities, *i*.*e*. involved in transporting the ions outside the cell to maintain the proper pH in the cell [45]. In case of cellular components, chosen genes are related to transcription factor complex, cytoplasmic membrane-bounded vesicle, membrane-bounded vesicle, cytoplasmic vesicle, vesicle and nucleoplasm part (Fig 2A). It can be seen that the maximum number of the genes is related to vesicle and membrane, which is consistent with the detoxifying mechanism of metal ions available in Al stress condition, especially in sequestration by vacuole [46, 47]. Some of the selected genes present on membrane are found to be involved in transporting of metal ions outside the cell or to the vacuole to maintain pH and transmembrane proton gradient [48].

### Gene co-expression network analysis for Al stress in soybean

**Fig 3:**
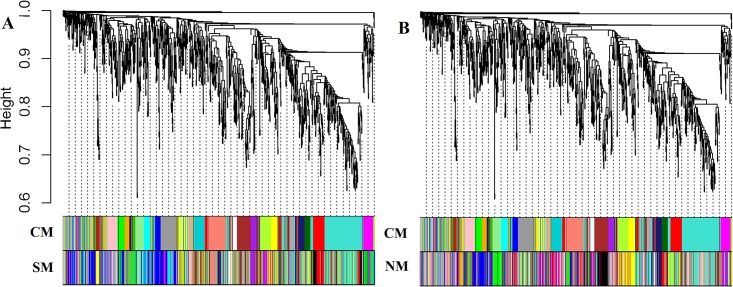
Clustering dendrogram of selected genes and gene modules under Al stress and control condition. The correspondence between Consensus Modules (CM) with modules under Stress (SM) (A) and control (NM) (B) conditions is represented.

**Fig 4:**
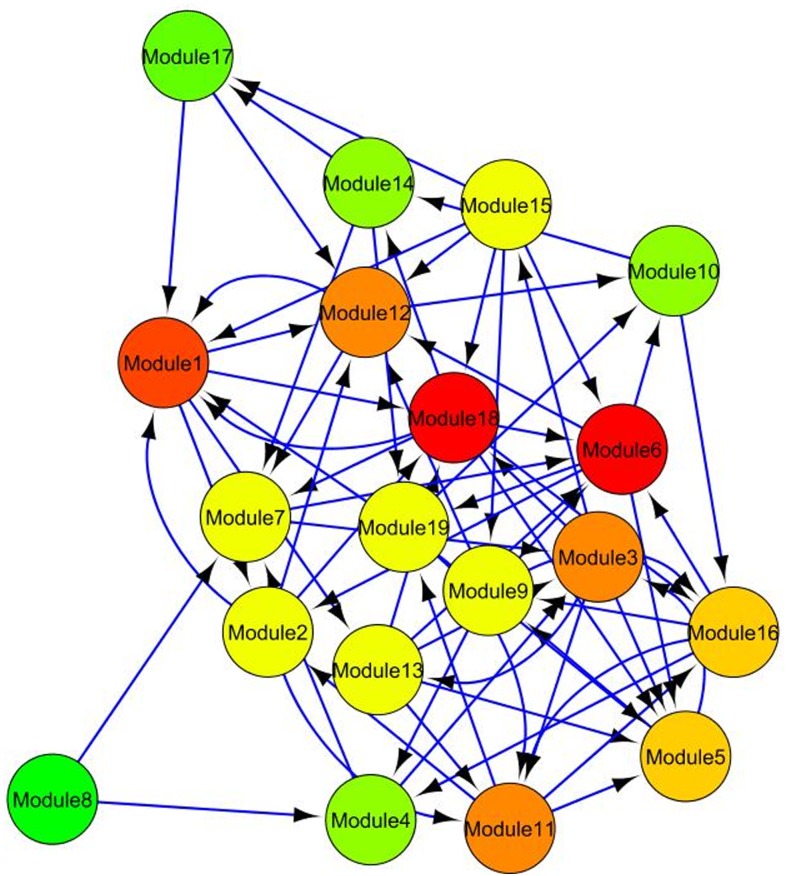
Module interaction network for gene modules under Al stress. The network consists of 19 nodes and 70 edges (regulatory relations). To remove the weak interaction among the modules, a threshold value for posterior probability is fixed at 0.2.

Using WGCNA, the selected 981 genes were divided into 19 and 18 modules (including grey colour module, which is the module of the non-modular genes) for Al stress and control conditions respectively (Fig 3). In both the cases, module represented by turquoise colour contains maximum number of genes, hence designated as the largest module for either condition. Based on the expression profiles of these selected genes for both Al stress and control conditions, 23 consensus modules (set of genes with similar co-expression patterns) were obtained. The matching of various modules for either condition with the consensus modules can be visualized from Fig 3 in terms of their colours. The extent of crosstalk between the various modules of consensus *vs*. control and consensus *vs*. stress conditions is given in S3 Fig. Further, the long length of branch in the dendrogram and high intensity of red colour in heat maps (S4 Fig) showed that the genes belong to same module have higher degree of co-expression as compared to genes present outside the module. The module memberships (number of genes present) of each module and their underlying molecular functions under Al stress condition are given in Table 4. It is observed that, every module is significantly annotated with GO terms, except gene modules represented by green-yellow and grey colour (Table 4). So, it can be inferred that functions of genes present within these two modules are still largely unknown. Furthermore, 19 identified modules (including grey module) were used as functional unit to model the MIN. The posterior probabilities of each module interactions were computed (using *i*BMA algorithm) and used to construct the MIN for Al stress as shown in Fig 4. It is seen that modules 1 and 18 are interacting with most of the modules in the MIN (Fig 4). Thus, these modules along with their members may play a significant role in Al stress response in soybean.

**Table 4:** List of gene modules along with their gene and hub gene memberships under Al stress condition.

SN	Module	G	AO	HG	UHG	Molecular Functions
1	Black	40	25	11	4	Monooxygenase activity, iron ion binding, heme binding, tetrapyrrole binding, oxidoreductase activity, cation binding ion binding,transition metal ion binding
2	Blue	137	68	0	0	Protein kinase activity, kinase activity, phosphotransferase activity, alcohol group as acceptor
3	Brown	100	68	38	32	Iron ion binding, hydrolase activity, acting on ester bonds, metal ion binding, cation binding, ion binding, transcription factor activity, DNA binding, protein kinase activity, phosphotransferase activity, transition metal ion binding, oxidoreductase activity, kinase activity
4	Cyan	29	23	0	0	Metal ion binding, cation binding, ion binding, transition metal ion binding, nucleic acid binding
5	Green	58	32	0	0	Protein kinase activity, phosphotransferase activity, protein serine /threonine kinase activity, protein tyrosine kinase activity, kinase activity
6	Green-yellow	33	17	3	3	Unknown
7	Grey	9	4	0	0	Unknown
8	Grey60	21	11	0	0	Binding
9	Light cyan	23	13	1	1	Binding
10	Light-green	16	11	0	0	Catalytic activity
11	Magenta	35	16	7	6	Hydrolase activity
12	Midnight-blue	24	11	5	3	Catalytic activity Binding
13	Pink	37	18	0	0	Nucleotide binding, ATP binding, adenyl ribonucleotide binding, purine nucleoside binding, nucleoside binding, adenyl nucleotide binding
14	Purple	34	20	0	0	Adenyl ribonucleotide binding adenyl nucleotide binding purine nucleoside binding, nucleoside binding, purine ribonucleotide binding, ribonucleotide binding, nucleotide binding
15	Red	54	28	20	2	Oxidoreductase activity
16	Salmon	31	15	0	0	Hydrolase activity, nucleotide binding
17	Tan	31	19	12	8	Hydrolase activity
18	Turquoise	185	106	86	45	Primary active transmembrane transporter activity, zinc ion, binding protein kinase activity, ATPase activity, cation transmembrane transporter activity, transition metal ion binding, metal ion binding, active transmembrane transporter activity, phosphotransferase activity, ATPase activity, cation binding, ion binding, ion transmembrane transporter activity, transferase activity, kinase activity
19	Yellow	84	49	45	26	Oxidoreductase activity
	Total	981	554	228	130	

SN: Serial number of module; grey module: genes which do not belong to any module are shown with grey colour; Module: module represented by colours; G: Number of genes belongs to the modules; AO: Number of *Arabidopsis* orthologs genes belong to each module; HG: Number of hub genes belong to each module; UHG: number of hub genes unique to stress

### Performance analysis of proposed hub gene detection approach

Based on the existing approach (*i*.*e*. WGS alone), 39.05% and 36.91% genes in the GCN are detected as hub genes in soybean for stress and control conditions respectively (Table 5). Thus, large proportions of genes are identified as hub genes in the GCN based on the existing approach, which contradicts the scale free property of biological networks (as GCN is a scale free network) [20, 36]. Similar findings are observed for salinity and cold stresses in rice (Table 5). However, in case of proposed approach (computing *p-values*) only 23.24% and 19.14% of genes in the GCN are found to be hub genes in soybean (for *p value* < 1E-10) for stress and control situations respectively (Table 5). Moreover, the number of hubs can be further reduced by decreasing the level of significance in the proposed approach. This indicates that the proposed approach will be able to identify relatively small subset of genes as hubs in the GCN *i*.*e*. fewer WGS are statistically significant. Similar results were obtained for the salinity and cold stresses in rice (Table 5).

**Table 5:** Comparison of proposed and existing approach in terms of predicted hub genes.

	Existing Approach		Proposed Approach			
Data sets	# HG	% HG	*p* value < 1E-5		*p* value < 1E-10	
			# HG	% HG	# HG	% HG
Salinity stress in rice						
Rice (Salinity stress)	214	38.49	187	33.63	165	29.66
Rice (Control)	229	41.19	208	37.41	180	32.36
Al stress in soybean						
Soybean (Al stress)	383	39.05	331	33.74	228	23.24
Soybean (Control)	362	36.91	285	29.05	187	19.14
Cold stress in rice						
Rice (Cold stress)	301	46.3	265	40.7	234	36
Rice (Control)	242	37.23	208	32	162	24.09

# HG: Number of hub genes;

% HG: Percentage of hub genes in the gene co-expression network;

Two thresholds for *p value* are taken as 1E-5 and 1E-10

**Fig 5:**
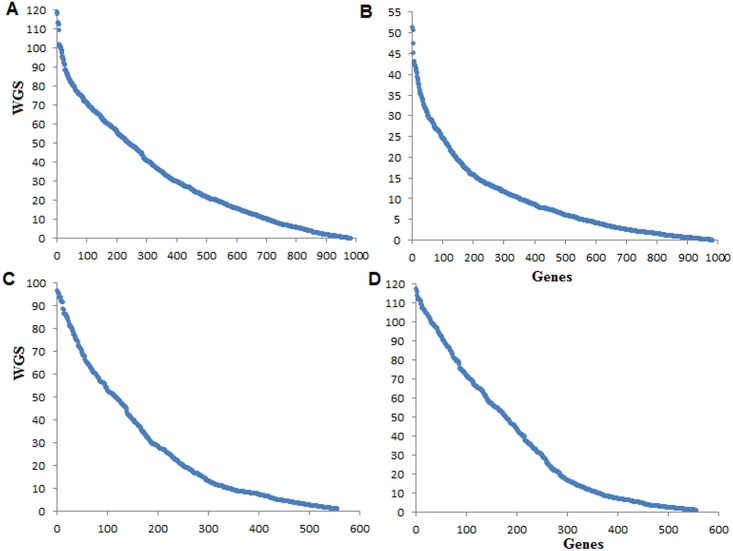
Distribution of WGS in complete networks under stress and control conditions. The distributions of WGS of genes in GCNs for Al stress (A) and control (B) conditions in soybean are shown. The distributions of WGS of genes in GCNs for salinity stress (C) and control (D) conditions in rice are shown. For all these cases, the distributions are heavy tailed.

**Fig 6:**
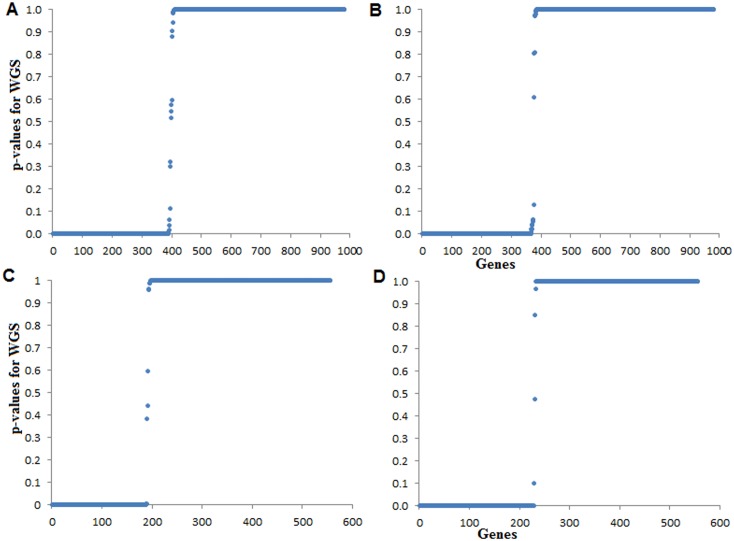
Distribution of *p-values* under stress and control conditions. The distributions of *p-values* of genes in GCNs for Al stress (A) and control (B) conditions in soybean are shown. The distributions of *p-values* of genes in GCNs for salinity stress (C) and control (D) conditions in rice are shown. Genes with low *p-values* represent highly interacting genes in the GCN.

The distributions of WGS (*i*.*e*. are heavy right tailed distributions) contain lower and upper values, which are not much discriminated between low and high connection degree of genes (Fig 5). On the contrary, from the distribution of *p-values*, the genes with high connection degrees are well separated from that of low connection degrees in the GCN (Fig 6). In other words, the distinction between statistically strongly and weakly connected genes in the GCN can be better seen from Fig 6 than Fig 5. Further, the results obtained from the proposed DHGA approach for two contrasting conditions (stress *vs*. control) are given in Table 6. The DHGA approach allowed grouping of 187 (stress) and 208 (control) hub genes of rice in the GCN into HHG (141), UHG to salinity stress (46) and UHG to control (67) (Table 6). Similar interpretations can be made for Al stress in soybean and cold stress in rice.

**Table 6:** Groups of hub genes predicted using DHGA approach.

Data	# Housekeeping hub	#UHG stress	#UHG control	# Non hub	# Total genes
Soybean (Al stress *vs*. control)	98	130	89	566	981
Rice (Salt stress *vs*. control)	141	46	67	161	556
Rice (Cold stress *vs*. control)	124	141	84	177	650

#Housekeeping Hub: Number of hub genes common to stress and control; #UHG stress: Number of hub genes unique to stress; #UHG control: Number of hub genes unique to control; #Non hub: Number of genes which are not hub gene in the GCN; #Total genes: Total number of genes in GCN

### Differential hub gene analysis for Al stress condition in soybean

**Fig 7:**
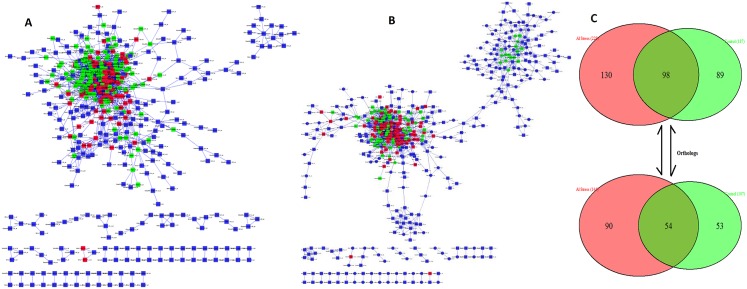
Gene Co-expression Networks for two differential conditions in soybean. The GCNs are constructed for Al stress (A) and control (B) conditions respectively. The nodes with red colors represent the housekeeping hub genes, green color nodes represent UHG and blue color nodes represent the non-hub genes. (C) Venn diagram of hub genes in the GCNs constructed under Al stress (A) and control (B) conditions in soybean. The number of orthologous genes found in *Arabidopsis* corresponding to unique and common hub genes in soybean is also shown.

Following the above approach, 228 and 187 genes were identified as hub genes whose *p-values* were ≤ 1E-10 for Al stress and control conditions of soybean respectively (Table 6). From the DHGA result, it is seen that 98 hub genes are common whereas 130 and 89 hub genes are unique for Al stress and control conditions respectively (Fig 7C). The mapping of the HHG and UHGs in soybean to *Arabidopsis* genome leads to the identification of corresponding *Arabidopsis* orthologs genes (Fig 7C). The GCNs constructed for these two differential conditions (Al stress *vs*. control) in soybean along with the positions of hub genes and UHGs are shown in Fig 7.

The functional analysis of the selected hub genes under Al stress revealed their associated cellular mechanisms. From the GO analysis (in cellular components), it is observed that most of the hub genes are present in plastid, vacoule, membrane bounded vacuole, cytoplasmic vacuole (Fig 2B), mainly responsible for pumping out ions from the cell. In MF category, majority of the hub genes were found to be involved in nucleic acid, cation ion, metal ion and zinc ion binding activities (Fig 2B), which may be responsible for fixing metal ions. Further, a large portion of the hub genes were found to be responsible for nitrogen compound metabolic process, response to stress and chemical stimulus, defense response and signal transduction under biological process category.

The module membership of the hub genes as well as UHG under Al stress showed that most of the hub genes under Al stress condition belong to turquoise (86), yellow (45) and brown (38) modules (Table 4). Similarly, out of 130 UHG, mainly 45, 32 and 26 are the members of turquoise, brown and yellow colour modules respectively. Interestingly, it can be seen that the blue colour module contains the second highest number of selected genes (137), but have no hub genes, while the brown colour module is the third largest module (100 genes) contains 38 hub genes out of which 32 are UHG for Al stress. Further, brown colour module is found to be associated with various important functions like ion binding, redox activity, kinase activity, phosphortransferase activity (Table 4), which are important for the abiotic stress response in plants [45]. From the molecular biology point of view, the brown colour module along with its members seems to be very important for breeding Al stress resistant varieties. A brief description about hub genes and UHG for Al stress condition is provided in S2 and S3 Tables respectively.

## Discussion

The proposed Boot-SVM-RFE technique was found to be superior for selection of informative genes from high dimensional gene expression data. This approach is also advantageous over classical gene selection techniques like t-test and F-score, as it does not require any distributional assumptions about the data. In this technique, a *p-value* was assigned to each gene and genes with lower *p-values* were considered as informative for the particular condition/trait under investigation. The selection of informative genes based on *p-values* is scientific as well statistically meaningful to experimental biologists as compared to other techniques. Further, the bootstrap procedure used in this technique was expected to remove the spurious associations of the genes with their classes. The comparative analysis showed that the Boot-SVM-RFE performed better than existing techniques *i*.*e*. SVM-RFE, t-score, F-score, RF and IG in terms of CA. Besides, its performance can be considered as robust due to the lower CV values in CA for all window sizes.

The proposed statistical approach for hub gene identification allowed the ranking and selection of candidate hub genes in the GCN, based on an assessment of the statistical significance of the gene connections. This was done with a randomized resampling based procedure where statistical significance values were calculated based on the NP test, which does not require Gaussian assumptions of data. Further, genes with lower *p-values* represent highly connected genes in the GCN and thus designated as hub genes. Moreover, the randomisation procedure used in this approach allows one to test, whether the observed gene connectivity is greater than expected gene connectivity value by chance (*i*.*e*. rejection of null hypothesis of random association). This was also able to remove the spurious association among genes, as these associations are measured on the basis of PCC. It seems to be more statistically convincing to select hub genes based on *p-values* rather than WGS alone, because in comparison to WGS, the *p-values* provides a reliable measure of gene connectivity based on a statistical criterion (lower *p-value* indicates high gene connectivity and *vice-versa*). Further, the detected hub genes tend to have higher connection degrees and are widely separated from the genes with low connection degrees in the GCN. Moreover, based on this approach a few as well as important genes were identified as hubs in the GCN as compared to existing approach, which is in accordance with the scale free property of biological networks.

Using DHGA approach, genes in the GCN were grouped into various categories *like* HHG, non-hubs, UHG for stress and control based on the computed *p-values* for these two contrasting conditions. These identified hub genes may be considered as biomarkers for further studies, including analysis of their involvement in diverse cellular mechanisms. Further, the HHG can be used for the maintenance of basal cellular functions that are essential for the existence of a cell [49], whereas, UHG can be used in stress response engineering in crops for developing stress tolerant cultivars.

Understanding Al stress response mechanism in soybean is of paramount importance for plant breeders to develop Al stress tolerant cultivars. In public domain databases, there are few samples available related to Al stress in soybean, which have been generated over varying experimental conditions by multiple studies. Thus, meta-analysis was performed to combine these datasets and the meta-data was used for further statistical analysis. Then, developed techniques were applied to identify the responsible genes to understand stress response mechanism in this crop. It has been reported that there are two main processes involved in Al stress response in plants (i) exclusion of Al ions from root cells and (ii) detoxification of Al ions in the plant cells [50]. Some selected genes were found to be involved in transporting of metal ions outside the cell, which might be associated with the first process. The function like redox activity related to electron transport under chemical reactions that balances the charges during metallic ions transport [45], might be associated with the second process. The redox activity might also be related to ROS generation that is produced in plants in response to Al stress. Further, ROS also seriously disrupts normal metabolism of cell through peroxidation of lipids [51], proteins and nucleic acids [52]. The increased redox activity is consistent with activation of the anti-oxidative enzymes such as catalase, ascorbate peroxidase and guaiacol peroxidase under abiotic stress condition [53]. The activities under BP taxonomy like cellular glucan and cellular amino acid metabolic processes are known to increase in plants in response to various abiotic stresses [54] and other reported biological processes need to be studied in the context of Al toxic stress. The role of phosphotranferase activity in conferring tolerance against abiotic stresses *like* drought and salt in rice and *Arabidopsis* are well established [55]. The role of stress induced organic acid synthesis in conferring Al tolerance in higher plants are also well reported [56]. These processes might be related to detoxification of Al ions, which occurs rapidly after exposure to Al stress in plants [57].

### dhga R Software package

In order to facilitate the use of proposed hub gene detection and DHGA approaches, we have developed an R software package which includes dhga R package accompanying documentation and model real data examples. This package can be freely downloaded from https://cran.r-project.org/web/packages/dhga. This software is capable of providing weighted adjacency matrix, edge list, node list for constructing GCN, *p-values* for gene connections along with weighted gene scores, *etc*. It also able to identify hub genes and perform differential hub analysis in the GCN based on the proposed approaches. Also the outputs provided by this can be directly used as inputs for gene co-expression networks construction software like Cytoscape, Visant, *etc*. However, it is difficult to construct GCN using this software package on R-platform.

## Conclusions

This investigation has some contributions to gene co-expression analysis. First, a statistically sound Boot-SVM-RFE gene selection technique was proposed for the selection of informative genes from high dimensional gene expression data. Second, a new statistical approach was proposed for the identification of hub genes in a GCN. Third, the proposed DHGA approach may be used to group genes in the GCN into various categories based on their gene connectivity. Fourth, a statistical modelling approach was employed to find the interaction among the gene modules. Moreover, the proposed Boot-SVM-RFE and DHGA approach can be used for other case *vs*. control genomic studies including NGS expression study. This study also throws some light to understand the mechanism of Al stress response in soybean and some key important genes were reported. Moreover, functional enrichment analysis of these key genes revealed their associated intracellular functions under Al stress. This information revealed in this study on various molecular mechanisms *like* biosynthesis of secondary metabolites and stress specific roles of certain plant products may be useful for mitigation of Al stress in plants, particularly in soybean. These identified genes can act as potential targets for bio-engineering of Al toxic stress response in soybean.

## Supporting Information

S1 Document Descriptions about the GEO series and selected GEO samples for Al stress. (DOCX)

S1 Fig Heat map of correlation among selected microarray samples. It can be seen that the correlation is ≥ 0.9 and hence the samples are said to be homogeneous though they were generated across different experimental conditions.
(TIF)

S2 Fig Analysis of network topology for gene co-expression network. Here, Y-axis indicates scale-free fit index (model fit value) and X-axis represents various soft-thresholding powers. The red line indicates soft power at which the scale-free fit index cut-off value 0.85 and mean connectivity value 40 is reached.
(TIF)

S3 Fig Crosstalk between the modules for consensus, Al stress and control conditions. The extent of crosstalk between the Consensus Modules (CM) and modules found under stress (SM) and control (NM) condition are shown in matrices form. Each row of the Table corresponds to modules under individual condition (labeled by color names as well as text along with the number of genes in the modules), and column corresponds to consensus modules. Numbers in the Table indicate gene counts in the intersection of the corresponding modules. The figures in various colors in the Table showed the highest values.
(TIF)

S4 Fig Dendrograms and heatmaps of selected informative genes divided into tightly co-expressed gene modules under Al stress and control condition. The heat map depicts the correlations among the 981 genes detected by gene selection plot from microarray gene expression profiling under stress (A) and control (B) conditions. The intensity of deep red colour in the heat map shows the strong correlation among genes present in the module.
(TIF)

S1 Table 981 genes selected by gene selection plot. (XLSX)

S2 Table List of identified 228 hub genes under Al toxic stress along with their *Arabidopsis* orthologs. (XLSX)

S3 Table List of identified 130 unique hub genes under Al toxic stress condition. (XLSX)

S4 Table List of 45 identified unique hub genes in the largest module. (XLSX)
