# Supplementary material for: Statistical Approaches for Gene Selection, Hub Gene Identification and Module Interaction in Gene Co-Expression Network Analysis: An Application to Aluminum Stress in Soybean (Glycine max L.)
Source: PLoS One. 2017 Jan 5;12(1):e0169605. doi: 10.1371/journal.pone.0169605 (PMC5215982; doi:10.1371/journal.pone.0169605)
Supplement: S1 Document — (DOCX) [file pone.0169605.s001.docx]

**DOC S1 Descriptions about the microarray samples**

We obtained soybean microarray data from Gene Expression Omnibus with platform GPL 4592 (<http://www.ncbi.nlm.nih.gov/geo/query/acc.cgi?acc=GPL4592>) which contains 3855 samples and 56 series. Among these datasets, there are few samples pertained to mainly three experiments available to study the Aluminium (Al) stress in soybean. We selected the datasets GSE18423, GSE18517 and GSE18518 generated by Duressa et al. (2010^a^, 2010^b^ and 2011) to study the Al stress response mechanism in Soybean through meta-analysis. The data were generated from Affymetrix Soybean Genome Array (GPL 4592 in GEO), which contains 37500 probes and each probe corresponded to an individual gene. The detail descriptions about the samples under Al stress are given in Table 1.

**Table 1** Descriptions about microarray samples used in this study

| **Experiment** | **Sample** | **Stress Type** | **Replication** | **Time** | **Sample Labels** | **References** |
| --- | --- | --- | --- | --- | --- | --- |
| **GSE18423** | GSM459249 | Control | 1 | 2 | -1 | Duressa et al. 2010a |
| **GSE18423** | GSM459250 | Control | 2 | 2 | -1 | Duressa et al. 2010a |
| **GSE18423** | GSM459251 | Control | 1 | 12 | -1 | Duressa et al. 2010a |
| **GSE18423** | GSM459252 | Control | 2 | 12 | -1 | Duressa et al. 2010a |
| **GSE18423** | GSM459253 | Control | 3 | 12 | -1 | Duressa et al. 2010a |
| **GSE18423** | GSM459254 | Control | 1 | 48 | -1 | Duressa et al. 2010a |
| **GSE18423** | GSM459255 | Control | 2 | 48 | -1 | Duressa et al. 2010a |
| **GSE18423** | GSM459256 | Control | 3 | 48 | -1 | Duressa et al. 2010a |
| **GSE18423** | GSM459257 | Control | 1 | 72 | -1 | Duressa et al. 2010a |
| **GSE18423** | GSM459258 | Control | 2 | 72 | -1 | Duressa et al. 2010a |
| **GSE18423** | GSM459259 | Control | 3 | 72 | -1 | Duressa et al. 2010a |
| **GSE18423** | GSM459260 | Stress | 1 | 2 | 1 | Duressa et al. 2010a |
| **GSE18423** | GSM459261 | Stress | 2 | 2 | 1 | Duressa et al. 2010a |
| **GSE18423** | GSM459262 | Stress | 1 | 12 | 1 | Duressa et al. 2010a |
| **GSE18423** | GSM459263 | Stress | 2 | 12 | 1 | Duressa et al. 2010a |
| **GSE18423** | GSM459264 | Stress | 3 | 12 | 1 | Duressa et al. 2010a |
| **GSE18423** | GSM459265 | Stress | 1 | 48 | 1 | Duressa et al. 2010a |
| **GSE18423** | GSM459266 | Stress | 2 | 48 | 1 | Duressa et al. 2010a |
| **GSE18423** | GSM459267 | Stress | 3 | 48 | 1 | Duressa et al. 2010a |
| **GSE18423** | GSM459268 | Stress | 1 | 72 | 1 | Duressa et al. 2010a |
| **GSE18423** | GSM459269 | Stress | 2 | 72 | 1 | Duressa et al. 2010a |
| **GSE18423** | GSM459270 | Stress | 3 | 72 | 1 | Duressa et al. 2010a |
| **GSE18517** | GSM461271 | Control | 1 | 2 | -1 | Duressa et al. 2011 |
| **GSE18517** | GSM461272 | Control | 2 | 2 | -1 | Duressa et al. 2011 |
| **GSE18517** | GSM461273 | Control | 1 | 12 | -1 | Duressa et al. 2011 |
| **GSE18517** | GSM461274 | Control | 2 | 12 | -1 | Duressa et al. 2011 |
| **GSE18517** | GSM461275 | Control | 3 | 12 | -1 | Duressa et al. 2011 |
| **GSE18517** | GSM461276 | Control | 1 | 48 | -1 | Duressa et al. 2011 |
| **GSE18517** | GSM461277 | Control | 2 | 48 | -1 | Duressa et al. 2011 |
| **GSE18517** | GSM461278 | Control | 3 | 48 | -1 | Duressa et al. 2011 |
| **GSE18517** | GSM461279 | Control | 1 | 72 | -1 | Duressa et al. 2011 |
| **GSE18517** | GSM461280 | Control | 2 | 72 | -1 | Duressa et al. 2011 |
| **GSE18517** | GSM461281 | Control | 3 | 72 | -1 | Duressa et al. 2011 |
| **GSE18517** | GSM461282 | Stress | 1 | 2 | 1 | Duressa et al. 2011 |
| **GSE18517** | GSM461283 | Stress | 2 | 2 | 1 | Duressa et al. 2011 |
| **GSE18517** | GSM461284 | Stress | 1 | 12 | 1 | Duressa et al. 2011 |
| **GSE18517** | GSM461285 | Stress | 2 | 12 | 1 | Duressa et al. 2011 |
| **GSE18517** | GSM461286 | Stress | 3 | 12 | 1 | Duressa et al. 2011 |
| **GSE18517** | GSM461287 | Stress | 1 | 48 | 1 | Duressa et al. 2011 |
| **GSE18517** | GSM461288 | Stress | 2 | 48 | 1 | Duressa et al. 2011 |
| **GSE18517** | GSM461289 | Stress | 3 | 48 | 1 | Duressa et al. 2011 |
| **GSE18517** | GSM461290 | Stress | 1 | 72 | 1 | Duressa et al. 2011 |
| **GSE18517** | GSM461291 | Stress | 2 | 72 | 1 | Duressa et al. 2011 |
| **GSE18517** | GSM461292 | Stress | 3 | 72 | 1 | Duressa et al. 2011 |
| **GSE18517** | GSM461293 | Control | 1 | 2 | -1 | Duressa et al. 2011 |
| **GSE18517** | GSM461294 | Control | 2 | 2 | -1 | Duressa et al. 2011 |
| **GSE18517** | GSM461295 | Control | 1 | 12 | -1 | Duressa et al. 2011 |
| **GSE18517** | GSM461296 | Control | 2 | 12 | -1 | Duressa et al. 2011 |
| **GSE18517** | GSM461297 | Control | 3 | 12 | -1 | Duressa et al. 2011 |
| **GSE18517** | GSM461298 | Control | 1 | 48 | -1 | Duressa et al. 2011 |
| **GSE18517** | GSM461299 | Control | 2 | 48 | -1 | Duressa et al. 2011 |
| **GSE18517** | GSM461300 | Control | 3 | 48 | -1 | Duressa et al. 2011 |
| **GSE18517** | GSM461301 | Control | 1 | 72 | -1 | Duressa et al. 2011 |
| **GSE18517** | GSM461302 | Control | 2 | 72 | -1 | Duressa et al. 2011 |
| **GSE18517** | GSM461303 | Control | 3 | 72 | -1 | Duressa et al. 2011 |
| **GSE18517** | GSM461304 | Stress | 1 | 2 | 1 | Duressa et al. 2011 |
| **GSE18517** | GSM461305 | Stress | 2 | 2 | 1 | Duressa et al. 2011 |
| **GSE18517** | GSM461306 | Stress | 1 | 12 | 1 | Duressa et al. 2011 |
| **GSE18517** | GSM461307 | Stress | 2 | 12 | 1 | Duressa et al. 2011 |
| **GSE18517** | GSM461308 | Stress | 3 | 12 | 1 | Duressa et al. 2011 |
| **GSE18517** | GSM461309 | Stress | 1 | 48 | 1 | Duressa et al. 2011 |
| **GSE18517** | GSM461310 | Stress | 2 | 48 | 1 | Duressa et al. 2011 |
| **GSE18517** | GSM461311 | Stress | 3 | 48 | 1 | Duressa et al. 2011 |
| **GSE18517** | GSM461312 | Stress | 1 | 72 | 1 | Duressa et al. 2011 |
| **GSE18517** | GSM461313 | Stress | 2 | 72 | 1 | Duressa et al. 2011 |
| **GSE18517** | GSM461314 | Stress | 3 | 72 | 1 | Duressa et al. 2011 |
| **GSE18518** | GSM461315 | Stress | 1 | 12 | 1 | Duressa et al. 2010b |
| **GSE18518** | GSM461316 | Stress | 2 | 12 | 1 | Duressa et al. 2010b |
| **GSE18518** | GSM461317 | Stress | 3 | 12 | 1 | Duressa et al. 2010b |
| **GSE18518** | GSM461318 | Stress | 1 | 72 | 1 | Duressa et al. 2010b |
| **GSE18518** | GSM461319 | Stress | 2 | 72 | 1 | Duressa et al. 2010b |
| **GSE18518** | GSM461320 | Stress | 3 | 72 | 1 | Duressa et al. 2010b |
| **GSE18518** | GSM461327 | Stress | 1 | 12 | 1 | Duressa et al. 2010b |
| **GSE18518** | GSM461328 | Stress | 2 | 12 | 1 | Duressa et al. 2010b |
| **GSE18518** | GSM461329 | Stress | 3 | 12 | 1 | Duressa et al. 2010b |
| **GSE18518** | GSM461330 | Stress | 1 | 72 | 1 | Duressa et al. 2010b |
| **GSE18518** | GSM461331 | Stress | 2 | 72 | 1 | Duressa et al. 2010b |
| **GSE18518** | GSM461332 | Stress | 3 | 72 | 1 | Duressa et al. 2010b |

In Sample Labels, “-1” and “1” means the microarray sample belong to control and Al toxic stress conditions respectively

**References**

Duressa D, Soliman K, Chen D (2010a) Identification of aluminum responsive genes in Al-tolerant soybean line PI 416937. International Journal of Plant Genomics. doi: 10.1155/2010/164862

Duressa D, Soliman KM, Chen D (2010b) Mechanisms of magnesium amelioration of aluminum toxicity in soybean at the gene expression level. Genome 53 (10):787-97. PMID: 20962885

Duressa D, Soliman KM, Taylor RW, Chen D (2011) Gene expression profiling in soybean under aluminum stress: genes differentially expressed between Al-tolerant and Al-sensitive genotypes. American Journal of Molecular Biology 1: 156-173. doi: 10.4236/ajmb.2011.13016.
