# Supplementary figures and images for: Statistical Approaches for Gene Selection, Hub Gene Identification and Module Interaction in Gene Co-Expression Network Analysis: An Application to Aluminum Stress in Soybean (Glycine max L.)
Source: PLoS One. 2017 Jan 5;12(1):e0169605. doi: 10.1371/journal.pone.0169605 (PMC5215982; doi:10.1371/journal.pone.0169605)

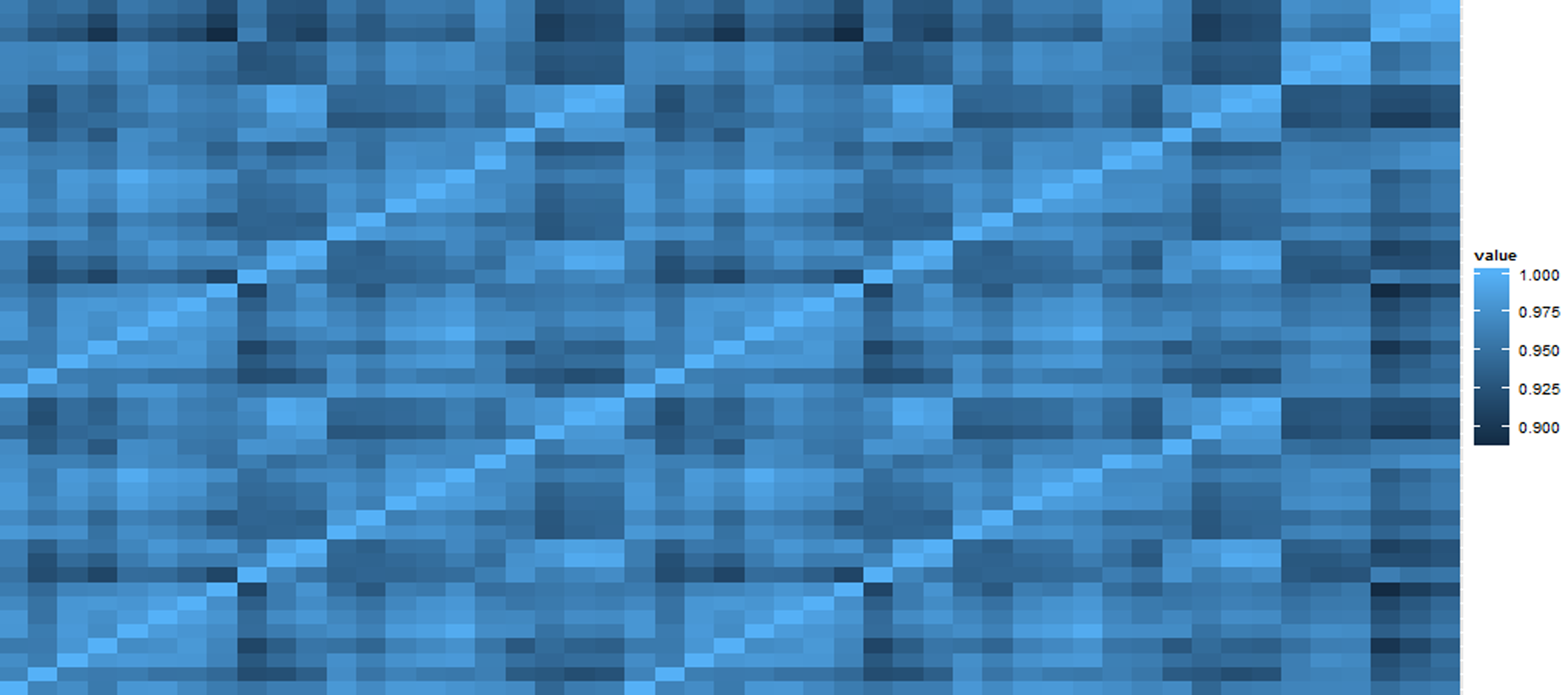

Supplement: S1 Fig — It can be seen that the correlation is ≥ 0.9 and hence the samples are said to be homogeneous though they were generated across different experimental conditions. (TIF) [file pone.0169605.s002.tif]

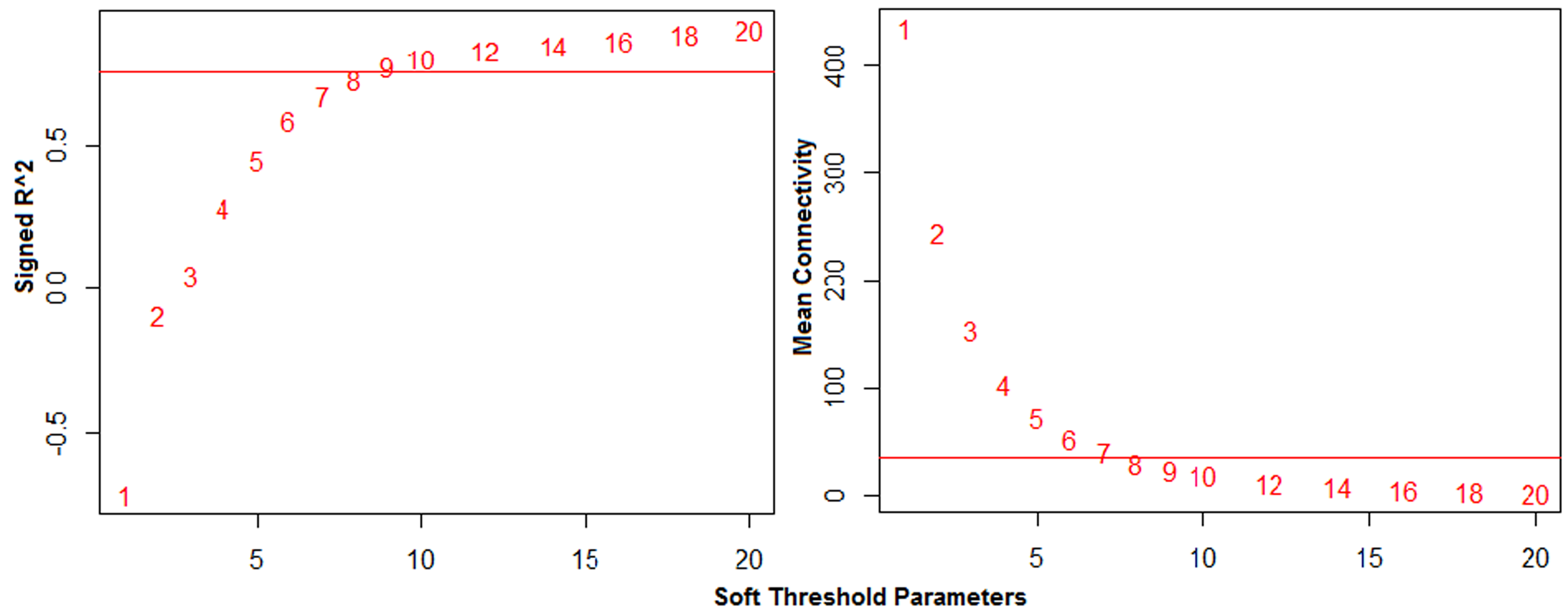

Supplement: S2 Fig — Here, Y-axis indicates scale-free fit index (model fit value) and X-axis represents various soft-thresholding powers. The red line indicates soft power at which the scale-free fit index cut-off value 0.85 and mean connectivity value 40 is reached. (TIF) [file pone.0169605.s003.tif]

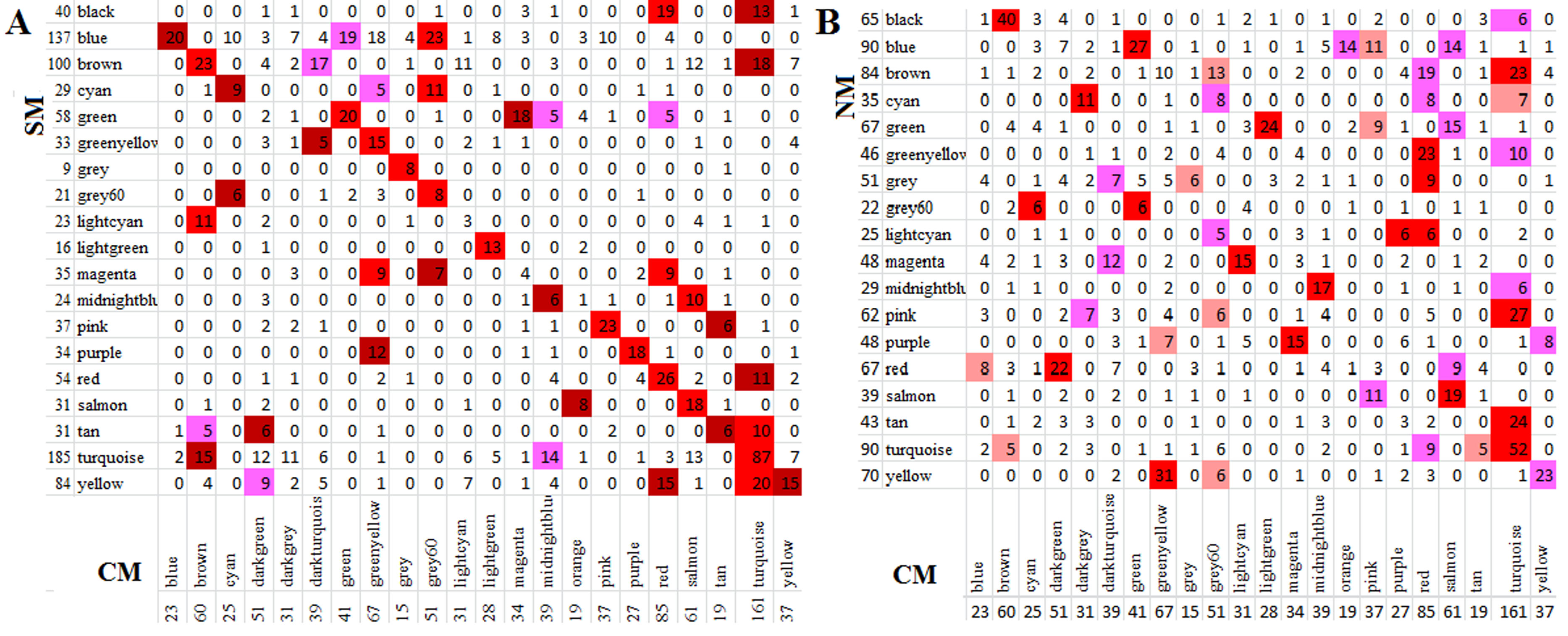

Supplement: S3 Fig — The extent of crosstalk between the Consensus Modules (CM) and modules found under stress (SM) and control (NM) condition are shown in matrices form. Each row of the Table corresponds to modules under individual condition (labeled by color names as well as text along with the number of genes in the modules), and column corresponds to consensus modules. Numbers in the Table indicate gene counts in the intersection of the corresponding modules. The figures in various colors in the Table showed the highest values. (TIF) [file pone.0169605.s004.tif]

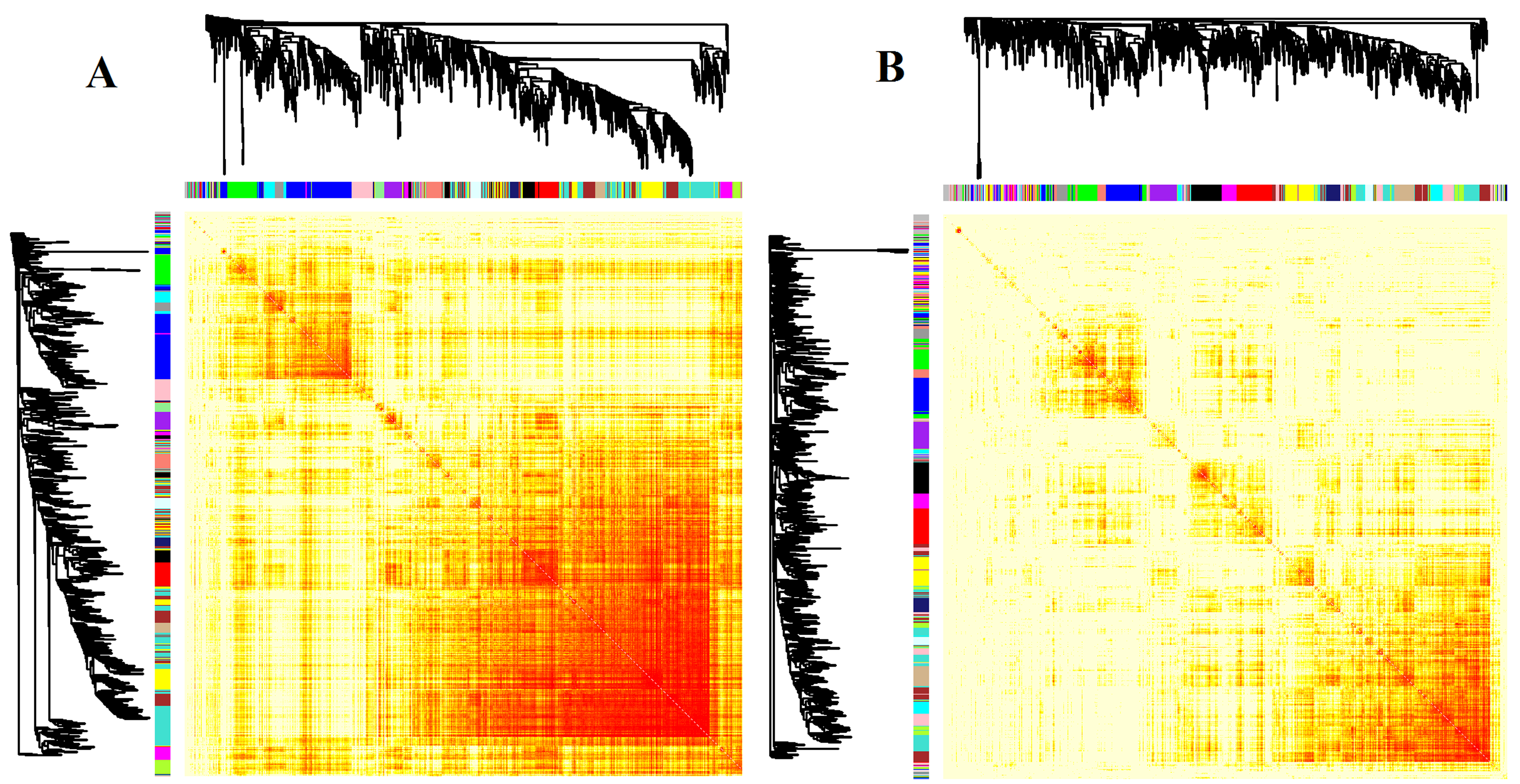

Supplement: S4 Fig — The heat map depicts the correlations among the 981 genes detected by gene selection plot from microarray gene expression profiling under stress (A) and control (B) conditions. The intensity of deep red colour in the heat map shows the strong correlation among genes present in the module. (TIF) [file pone.0169605.s005.tif]
